# Deep Brain Stimulation of the Habenula: Systematic Review of the Literature and Clinical Trial Registries

**DOI:** 10.3389/fpsyt.2021.730931

**Published:** 2021-08-17

**Authors:** Jürgen Germann, Manuel Mameli, Gavin J. B. Elias, Aaron Loh, Alaa Taha, Flavia Venetucci Gouveia, Alexandre Boutet, Andres M. Lozano

**Affiliations:** ^1^Division of Neurosurgery, Department of Surgery, University Health Network and University of Toronto, Toronto, ON, Canada; ^2^The Department of Fundamental Neuroscience, The University of Lausanne, Lausanne, Switzerland; ^3^INSERM, UMR-S 839, Paris, France; ^4^Sunnybrook Research Institute, Sunnybrook Health Sciences Centre, University of Toronto, Toronto, ON, Canada; ^5^Joint Department of Medical Imaging, University of Toronto, Toronto, ON, Canada

**Keywords:** habenula, deep brain stimulation, clinical trial, depression, obsessive-compulsive disorder, schizophrenia, bipolar disorder

## Abstract

The habenula is a small bilateral epithalamic structure that plays a key role in the regulation of the main monoaminergic systems. It is implicated in many aspects of behavior such as reward processing, motivational behavior, behavioral adaptation, and sensory integration. A role of the habenula has been indicated in the pathophysiology of a number of neuropsychiatric disorders such as depression, addiction, obsessive-compulsive disorder, and bipolar disorder. Neuromodulation of the habenula using deep brain stimulation (DBS) as potential treatment has been proposed and a first successful case of habenula DBS was reported a decade ago. To provide an overview of the current state of habenula DBS in human subjects for the treatment of neuropsychiatric disorders we conducted a systematic review of both the published literature using PUBMED and current and past registered clinical trials using ClinicalTrials.gov as well as the International Clinical Trials Registry Platform. Using PRISMA guidelines five articles and five registered clinical trials were identified. The published articles detailed the results of habenula DBS for the treatment of schizophrenia, depression, obsessive-compulsive disorder, and bipolar disorder. Four are single case studies; one reports findings in two patients and positive clinical outcome is described in five of the six patients. Of the five registered clinical trials identified, four investigate habenula DBS for the treatment of depression and one for obsessive-compulsive disorder. One trial is listed as terminated, one is recruiting, two are not yet recruiting and the status of the fifth is unknown. The planned enrollment varies between 2 to 13 subjects and four of the five are open label trials. While the published studies suggest a potential role of habenula DBS for a number of indications, future trials and studies are necessary. The outcomes of the ongoing clinical trials will provide further valuable insights. Establishing habenula DBS, however, will depend on successful randomized clinical trials to confirm application and clinical benefit of this promising intervention.

## Introduction

Neurological and psychiatric brain disorders emerge from the aberrant activity in brain circuits (1–4). Deep Brain Stimulation (DBS) employs precisely placed electrodes to deliver current to specific brain structures in order to modulate these dysfunctional circuits (1, 5). To date, well over 200,000 patients world-wide have been treated with DBS, most commonly for the management of movement disorders, such as Parkinson's disease (PD) (1). DBS offers advantages over other neuromodulatory treatments as it is non-lesional, reversible, and stimulation parameters can be adjusted as needed. The effectiveness of DBS depends upon appropriately and selectively stimulating and modulating the intended brain circuit(s) and is contingent on the selection of the optimal anatomical target and fine tuning the stimulation. For each DBS patient, stimulation parameters have to be individually optimized to maximize clinical benefits and minimize side-effects. This parameter optimization–or “programming”–, however, remains an empirical trial-and-error process that necessitates repeated clinic visits and is thus time- and resource-intensive (6) for both the patients and healthcare systems. A number of brain structures have been proposed as targets for DBS with multiple potential targets identified for most conditions (3, 7–12).

**Figure 1:**
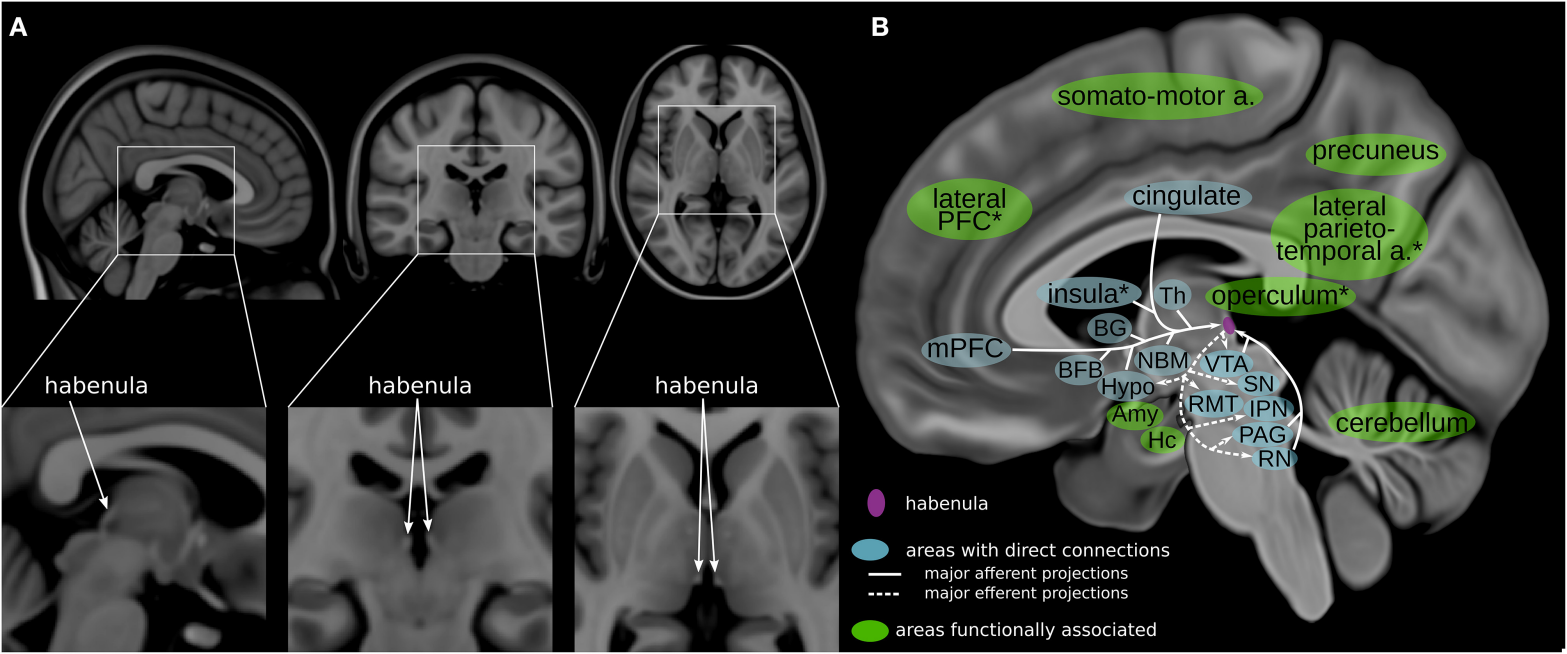
Habenula anatomy and connections. **(A)** The human habenula illustrated in orthogonal slices using the MNI template brain (MNI152b) (16). **(B)** Highlighting regions connected with the habenula (17–20) illustrated using a medial view of a high-resolution, high contrast template (21). The habenula is highlighted in purple. Areas with direct connections to the habenula are highlighted in blue; areas that are functionally associated with the habenula are highlighted in green. a, areas; Amy, amygdala; BFB, basal forebrain; BG, basal ganglia; Hc, hippocampus; Hypo, hypothalamus; IPN, interpeduncular nucleus; mPFC, medial prefrontal cortex; NBM, nucleus basalis of Meynert; PAG, periaqueductal gray; PFC, prefrontal cortex; RMT, rostromedial tegmental nucleus; RN, raphe nuclei; SN, substantia nigra; Th, thalamus; VTA, ventral tegmental area; *approximate/example position of the region.

The habenula (Hb) is a relatively new DBS target that has been proposed to treat various psychiatric disorders, including depression (7, 13). It is a small bilateral epithalamic structure located adjacent to the posterior commissure in humans (14, 15) (Figure 1A). Invasive studies using animal models have shown that the Hb has extensive direct connections with the hypothalamus, brainstem nuclei, basal ganglia and limbic areas–the stria medullaris being the main afferent and the fasciculus retroflexus the main efferent fiber bundle (Figure 1B; Table 1) (17–19, 25–28). Studies in humans using imaging and electrophysiological techniques have shown multiple additional cortical and cerebellar regions to be functionally connected to the habenula (Figure 1B; Table 1) (20, 22–24, 28). It plays a key role in controlling the dopaminergic, serotonergic and noradrenergic systems (25, 27, 29–32). The Hb thus has a unique position regulating the three main monoaminergic systems.

**Table 1:** Connectivity of the habenula.

**Structure**	**Type of connectivity**	**Model**	**Technique**
Amygdala	Functionally connected	Clinical	Functional MRI & electrophysiology (20, 22–24)
Basal forebrain	Direct afferent	Pre-clinical	Tracer (25)
Basal ganglia	Direct afferent	Pre-clinical	Tracer (25)
Cerebellum	Functionally connected	Clinical	Functional MRI (20, 22)
Cingulate cortex	Direct efferent	Pre-clinical	Tracer (26)
Hippocampus	Functionally connected	Clinical	Functional MRI & electrophysiology (20, 22, 23)
Hypothalamus	Direct afferent + efferent	Pre-clinical	Tracer (25, 27)
Insula	Direct efferent	Pre-clinical	Tracer (26)
Intrapeduncular nucleus	Direct efferent	Pre-clinical	Tracer (27)
Lateral parieto-temporal areas	Functionally connected	Clinical	Functional MRI (20, 22)
Lateral prefrontal cortex	Functionally connected	Clinical	Functional MRI (20, 22)
Medial prefrontal cortex	Direct efferent	Pre-clinical	Tracer (26)
Nucleus basalis of Meynert	Direct afferent	Pre-clinical	Tracer (25)
Operculum	Functionally connected	Clinical	Functional MRI (20, 22)
Periaqueductal gray	Direct afferent + efferent	Pre-clinical	Tracer (25, 27)
Precuneus	Functionally connected	Clinical	Functional MRI (20, 22)
Raphe nuclei	Direct afferent + efferent	Pre-clinical	Tracer (25, 27)
Rostromedial tegmental nucleus	Direct efferent	Pre-clinical	Tracer (27)
Somato-motor areas	Functionally connected	Clinical	Functional MRI (20, 22)
Substantia nigra	Direct efferent	Pre-clinical	Tracer (27)
Thalamus	Direct afferent	Pre-clinical	Tracer (25)
Ventral tegmental area	Direct afferent + efferent	Pre-clinical	Tracer (25, 27)

*MRI, magnetic resonance imaging*.

Evolutionarily preserved across vertebrae, the Hb can be divided into a medial and a lateral Hb. The medial Hb is composed mainly of glutamate producing neurons that exert influence over the serotonergic system and are involved in emotional response selection (28, 33–35). The lateral Hb (LHb) is composed mainly of glutamate producing neurons that control midbrain dopaminergic neurons and is involved in reward related behavior (17, 31). Pioneering research analyzing the role of the Hb in processing reward related information by Hikosaka and others demonstrated that the LHb plays a key role in controlling adaptive behaviors (17). Neuronal activity in the LHb diminishes upon presentation of rewards, while activity increases after the presentation of aversive stimuli or cues predicting them (36–39). A series of studies indicate that maladaptations within the LHb underlie behavioral symptoms of major depression. Indeed, several cellular, and synaptic adaptations are causally linked to LHb hyperactivity, which consequently drives the expression of anhedonia and behavioral despair, which are typical aspects of mood disorders (40–42). Playing a key role in the control of all three major monoaminergic transmitter systems, the Hb controls a wide range of behavior beyond reward processing and depressive symptoms. The habenula has been connected to social interaction, motivational behavior, behavioral adaptation, pain processing and sensory integration, motor activity, memory, sleep, and circadian rhythm (17–19, 22, 43–46).

**Figure 2:**
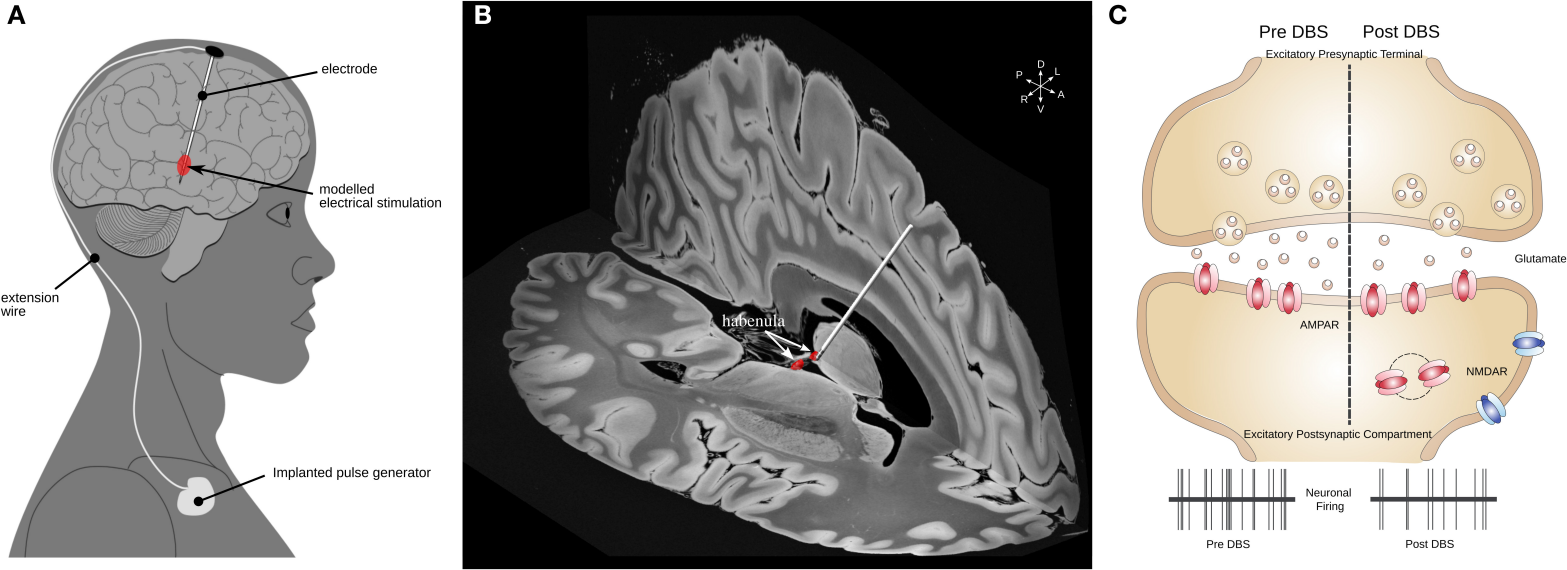
Habenular DBS and its mechanisms of action. **(A)** Schematic illustrating the components of a DBS system. **(B)** 3-D example image of a DBS electrode targeting the left Hb (red) illustrated in MNI152 space (16) using slices from a 100 micron resolution, FLASH 7 Tesla brain (48) electrode placement performed using Lead-DBS (49). **(C)** Mechanisms of action of DBS in LHb. A schematic of excitatory synapse is presented. Human-inspired DBS protocols in rodents lead to a reduction in glutamate release, which subsequently reduces the neuronal activity of LHb neurons (41, 50). DBS, deep brain stimulation; Hb, habenula; LHb, lateral habenula; MNI, Montreal Neurological Institute.

In a first seminal study, DBS within the Hb successfully ameliorated depressive symptoms in a patient where classic pharmacological treatment had failed (47). The components of an implanted DBS system and an example electrode targeting the Hb are illustrated in Figures 2A,B. These findings prompted a series of studies to assess whether DBS was similarly effective in animal models and to evaluate the underlying therapeutic mechanisms. Using a rodent model of depression named learned helplessness, an initial study reported an increased synaptic excitation onto LHb neurons concomitantly with increased neuronal activity of LHb cells with respect to control rats (41, 42). Adapted DBS-like electrodes were then inserted in the LHb, and high frequency stimulation, similar to that employed in humans, normalized the depressive-like state typical in learned helplessness rats (41). Importantly, DBS potently produced a time-locked collapse of glutamatergic transmission onto LHb neurons (41). This initial finding represented an initial indication that changes in cellular function in the LHb causally linked to depressive states, and that LHb-targeted DBS could represent a therapeutically-relevant strategy. In support of this, a different study employed early life stress to drive the emergence of depressive-like symptoms in adulthood that included defects in coping strategies and anhedonia (50). Mechanistically, the behavioral changes were associated with a reduction in the postsynaptic function of the metabotropic GABAb receptors (Figure 2C). The metabotropic GABAb receptor is a key cellular module for the maintenance of neuronal activity within the LHb (40). The reduction in GABAb function led to higher neuronal firing activity in LHb neurons (40, 50).

The authors used DBS-like electrodes in acute slices to show that this also causes a presynaptic reduction in glutamate release, as well as efficient reduction in neuronal firing rate assessed using *in vivo* recordings (50). Further, the use of DBS in behaving animals submitted to early life stress produced a normalization of the depressive-like state compared to non-stimulated animals (50). These studies not only unravel mechanisms of action of DBS in the LHb but support its use for therapeutically relevant interventions.

The potential of the Hb as a target for DBS goes beyond the context of depression. For example, DBS of LHb in rats reduced sucrose seeking and cocaine seeking behavior (51, 52) consistent with the putative role of Hb in addiction (17, 34, 53, 54). Furthermore, studies have demonstrated that the Hb plays a role in the pathophysiology of a number of neuropsychiatric disorders beyond depression and addiction such as schizophrenia (15, 55, 56), bipolar disorder (BD) (15, 33, 57–59), obsessive-compulsive disorder (OCD) (60, 61) and autism (62).

Given the multitude of potential therapeutic applications of Hb DBS, we conducted a systematic review of both the published human literature and the registered clinical trials to provide an overview of the current status of Hb DBS.

## Methods

**Figure 3:**
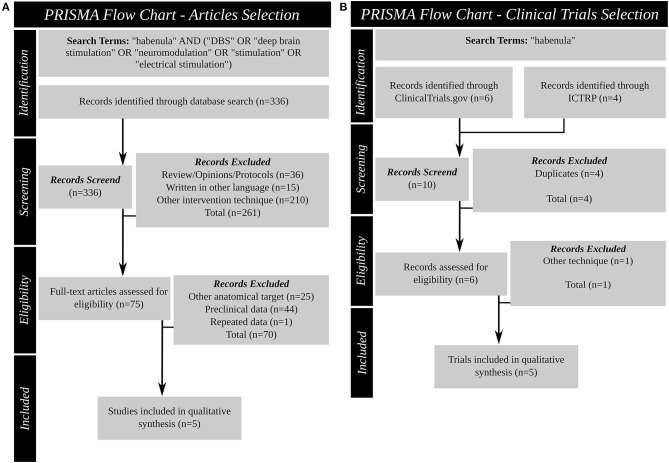
Systematic review PRISMA figures. **(A)**: Article search **(B)**: Clinical trial database search. DBS, deep brain stimulation; ICTRP, international clinical trials registry platform; PRISMA, Preferred Reporting Items for Systematic Reviews and Meta-Analyses.

This systematic review was performed according to PRISMA guidelines (Figure 3). In March 2021, a literature search was conducted for original articles using PubMed/MEDLINE with the following search term: “habenula” AND (“DBS” OR “deep brain stimulation” OR “neuromodulation” OR “stimulation” OR “electrical stimulation”). No restrictions were placed on the publication date. No duplicates were found. Articles written in languages other than English, protocols, reviews, and opinion pieces as well as articles describing techniques other than DBS were excluded. All relevant articles were selected for full-text review and had to meet the following inclusion and exclusion criteria: (I) inclusion (articles reporting on the clinical outcome of Hb deep brain stimulation in humans) and (II) exclusion (studies reporting preclinical data; deep brain stimulation targeting a different brain region; articles reporting a patient population described previously).

Also in March 2021 two publicly available clinical trial databases were queried for past and ongoing clinical trials using “habenula” as search term: ClinicalTrials.gov (https://clinicaltrials.gov/) provided by the US National Library of Medicine, and the International Clinical Trials Registry Platform (ICTRP; https://www.who.int/ictrp/en/; https://apps.who.int/trialsearch/) of the World Health Organization (WHO). All entries since inception of the databases were queried. Duplicates were excluded. All relevant trials were selected for review and had to meet the inclusion and exclusion criteria: (I) inclusion (deep brain stimulation targeting the Hb as primary intervention) and (II) exclusion (intervention other than DBS).

Studies and trials were separately screened (JG and AT) and disagreement was resolved by consensus.

## Results

Of the 336 items identified in the search, 36 were reviews, technical protocols or opinion pieces, 15 were not written in English, and 210 described an intervention other than DBS (e.g., optogenetic stimulation, neurochemical stimulation). The full text search of the remaining 75 studies 25 did not target the Hb, 44 described preclinical experiments and one study described data reported in an earlier manuscript. After screening, this systematic review identified five articles (Table 2) (47, 63–66) that satisfied the inclusion criteria. These articles reported the results of DBS targeting the Hb for four different indications: schizophrenia (63), treatment resistant major depressive disorder (TRD) (47, 65, 66), OCD (45) and BD (46).

**Table 2:** Articles selected.

**References**	Wang et al. (63)	Sartorius et al. (47)	Zhang et al. (64)	Zhang et al. (65)	Wang et al. (66)
**Study type**	Case series	Case study	Case study	Case study	Case study
***N* (m/f)**	2 (2/0)	1(0/1)	1(1/0)	1(1/0)	1(1/0)
**Age (avg, range)**	23.5 (21–26)	64	30	41	34
**Disorder**	Schizophrenia	TRD	OCD	BD	TRD
**Disease Duration (avg/range)**	6.5y (4–9)	46y	15y	21y	21y
**Medication**	Patient 1: olanzapine 15 mg/day from month 7 onwards (previous dose unknown); patient 2: que-tiapine 0.4 g/dayfrom month 4 onwards (previous dose unknown)	N/D	Fluvoxamine (100 mg/d); PRE-DBS: Benzhexol (2 mg/d);Olanzapine (7.5 mg/d)Magnesium Valproate (0.25 g/d)	N/D	N/D
**DBS procedure**	Bilateral	Bilateral	Bilateral	Bilateral	Bilateral
**DBS target**	Habenula	Lateral habenula	Habenula	Habenula	Lateral habenula
**Follow-up**	10 to 12 months	57 weeks	12 months	12 months	12 weeks
**% improvement (avg/range)**	11.1%(−9.5–31.7%)	100.00%	35.50%	100.00%	56.50%
**Positive clinical outcome ratio**	1/2	1/1	1/1	1/1	1/1
**Assessment tool**	PANSS	HAMD21	Y-BOCS	HAMD17	HAMD21
**Secondary outcome% improvement (avg/range)**	PANSS pos scale:-7.7%(-69.2–53.8%); PANSS neg scale: 13.7%(-4.3–31.7%); PANSS Gen Psychopathology: 14.35% (7.9–20.8%)	N/D	HAMD17: 36%; HARS: 31%; PSQI: −20%; EQ-5D-5L: 33%	MADRS: 100%; HARS: 87%; PSQI: 44%Plus COGSTATE, SF-36 and SDS with multiple sub-scales	MADRS: 66%; QIDS-SR: 63%; HARS: 65%; PSQI: 93%; HCL-32: 86%
**Side effects**	Acute: numbness, change of heart rate, pain, dizziness, eye closing, discomfort, feeling of heaviness, feeling of relaxation.	N/D	Acute: dizziness, numbness, nausea, flusteredness	Blurred vision temporary (high frequency stimulation)	Acute: dizziness, numbness, nausea, flusteredness
**Stimulation settings**	Patient 1: L (2.0 V, 60 us, 60 Hz); R (2.5 V, 60 μs, 60 Hz); patient 2: L (3.15 V, 80 us, 135 Hz); R (3.2 V, 60 μs, 135 Hz)	10.5V	L (1.6 V, 60 μs, 60 Hz); R (1.35 V, 60 μs, 60 Hz)	L+R (2.0 V, 60 μs, 60 Hz)	L (4.5 V, 90 μs, 160 Hz); R (2.5 V, 90 μs, 160 Hz)
**Observations**	At 6 months both patients showed positive treatment response, only 1 patient retained positive treatment response at 12 months	No acute antidepressant effect; interval to remission 4 months after switching to high frequency stimulation; stimulation location confirmed using FDG-PET; symptoms return when treatment accidentally discontinued	N/D	High frequency stimulation provided 46% improvement; low-frequency stimulation initiated in the fourth quarter with 100% improvement; functional connectivity compared to healthy controls at various time points	LFP results indicate decreased habenula activity with stimulation ON; magnitude of suppression associated with clinical improvements; power spectrum density decreases over time with treatment (acquired with stimulation OFF)

*BD, bipolar disorder; COGSTATE, computerized cognitive assessment tool; DBS, deep brain stimulation; EQ-5D-5L, 5-level EuroQol-5D; FDG-PET, fluorodeoxyglucose positron emission tomography; HAMD, Hamilton Depression Scale; HARS, Hamilton anxiety rating Scale; HCL-32, hypomania checklist; L, left; LFP, local field potentials; MADRS, Montgomery-Asberg Depression Rating Scale; N/D, not described; OCD, obsessive-compulsive disorder; PANSS, positive and negative symptom scale; PSQI, Pittsburgh sleep quality index; QIDS-SR, Quick Inventory of Depressive Symptomatology; R, right; SDS, Sheehan disability scale; SF-36, 36-item short survey; TRD, treatment resistant major depressive disorder; Y-BOCS, yale-brown obsessive compulsive scale*.

Table 3 outlines the evidence and rationale the studies provide for using Hb DBS as treatment in each of the various psychiatric conditions.

**Table 3:** Rationale for Habenula involvement in psychiatric disorders.

	**Depression**	**Bipolar disorder**	**Schizophrenia**	**Obsessive-compulsive disorder**
Evidence for involvement of habenula	Animal studies implicate the habenula in depressive-like behavior (45, 67). The LHB is found to be hyperactive and ablation of the habenula in animals alleviates depressive-like symptoms (33, 67) (proulx; Fakhoury). Altered habenula volume and function has been reported in human subjects (58, 68).	Altered habenula volume and function has been reported in human subjects (15, 58, 69). Animal models show involvement of LHB in mood disorders [see Depression].	Altered habenula volume and function has been reported in human subjects (15, 70, 71). Rodent models show LHB function important for guided decision making, and LHB hypoactivity associated with schizophrenia-like symptoms (45, 72). Antipsychotic drugs increase LHB activity in animal model (33)	Encoding and processing of aversive stimuli–processes dependent on the LHB–are impaired in OCD (36, 73, 74). Depressive symptoms are often present in OCD (75). Hb-DBS has previously been used to successfully treat depression (47)
Proposed habenula contribution to the psychiatric disorder and/or symptoms	LHB encodes negative motivational values and aversive outcomes associated with cues (37). Continuously hyperactive LHB causes depressive state (13, 47).	Hyperactive LHB causes depressive state (13, 47) [see Depression].	The habenula plays a key role in controlling the monoaminergic systems and these systems are disturbed in SZ (17, 76). Hypoactive LHB interferes with appropriate guidance of behavior and decision making (45).	Monoaminergic systems are disturbed in OCD and the habenula plays a key role in their control (17, 77).
Proposed mechanism of the beneficial effect of habenula DBS	DBS reduced LHB neuronal activity via GABAb receptors (40, 50).	DBS corrects habenula dysfunction (65) [see Depression].	DBS corrects habenula hypoactivity (63).	DBS corrects habenula dysfunction (64).

*DBS, Deep brain stimulation; LHB, Lateral Habenula; OCD, Obsessive-Compulsive Disorder; SZ, Schizophrenia*.

Four of the five were single case studies (47, 64–66), one was a small case series of two patients (63). Clinical changes ranged from −9.5 to 100% symptom improvement in the overall six patients treated, with positive clinical outcome (improvement ≧31.7%) reported in 5 patients. The patients ranged in age from 21 to 64 years, disease duration at time of surgery varied from 4 to 46 years, and five of the six were male. One patient dropped out after 18 weeks for non-medical reasons (47) and all other patients were observed for at least >10 months. In all cases reported, Hb DBS stimulation was delivered via bilateral electrodes using a monopolar configuration and a pulse-width of 60 μs. Frequency of stimulation spanned from 60 to 160 Hz and voltage ranged from 1.35 to 10.5V. Studies describe numerous side effects of acute stimulation, all of which were subsequently controlled by altering the stimulation parameters (e.g., lower voltage, change contact, change frequency).

In addition to published articles, the search found 10 trial entries in the two clinical trial databases. The search results of these clinical trial databases provide insight into the current research investigating the habenula as a DBS target. Four duplicates were excluded and the records revealed that one trial did not use Hb DBS. Therefore, five clinical trials (Table 4) (NCT03463590, NCT03347487, NCT03254017, NCT01798407, NCT03667872) were identified using Hb DBS for two different indications: TRD (NCT03347487, NCT03254017, NCT01798407, NCT03667872) and OCD (NCT03463590). One trial (NCT01798407) is a non-randomized trial using quadruple masking, the other four are open label trials. Two of the publications identified in the literature search are associated with two of the clinical trials found: the study of Wang and colleagues (66) with NCT03667872 and the work of Zhang and colleagues (65) with NCT03254017. One trial is currently recruiting, one has been terminated, two are not yet recruiting and the status of the last is unknown. Bilateral Hb DBS is planned in all five trials and planned enrollment ranges from 2 to 13 patients, or 6 to 13 in the trials not terminated. China is the country of origin of four, the United states of one trial. All trials plan to recruit adults (from 18 or 21 years to 65 or 70 years of age) of both sexes.

**Table 4:** Registered clinical trials of habenula DBS.

**NCT number**	**NCT03463590**	**NCT03347487**	**NCT03254017**	**NCT01798407**	**NCT03667872**
Link	https://ClinicalTrials.gov/show/NCT03463590	https://ClinicalTrials.gov/show/NCT03347487	https://ClinicalTrials.gov/show/NCT03254017	https://ClinicalTrials.gov/show/NCT01798407	https://ClinicalTrials.gov/show/NCT03667872
Title	Deep Brain Stimulation of the Bilateral Habenula for Treatment-Refractory Obsessive-Compulsive Disorder	DBS of the Habenula for Treatment- Resistant Major Depression	Remotely Programmed Deep Brain Stimulation of the Bilateral Habenula for Treatment- Resistant Major Depression: An Open Label Pilot Trial	DBS of the Lateral Habenula in Treatment-Resistant Depression	Efficacy and Safety of DBS in Patients With Treatment-Resistant Depression
Status	Unknown status	Recruiting	Terminated	Active, not recruiting	Not yet recruiting
Condition	OCD	TRD	TRD	TRD	TRD
Intervention	Device: Bilateral surgical implantation of DBS system to habenula	Procedure: Deep brain stimulation system implantation	Procedure: Bilateral surgical implantation of DBS system to Habenula Other: Follow-up Period	Device: Activa Tremor Control Sys (DBS Implant)Other: Randomized, staggered withdrawal phase	Procedure: Bilateral implantation of DBS system to Habenula
Outcome measures	Questionnaires: Y-BOCS II; OCI-R; HAMD; HAMA; WHO-BREF; SF-36 Neuropsychologic: Cogstate battery Imaging: fMRI	Questionnaires: HAMD; MADRS; YMRS; HAMA; GAF; C-SSRS; WHO-BREF; SF-36; BDI; PSQI; Q-LES-Q-SF; SDSNeuropsychologic:CANTAB tasksOthers:Brain activity; Side Effects	Questionnaires: MADRS; HAMD; HAMA; SF-36; WHO-BREF; YMRS; PSQI Neuropsychologic: Cogstate battery	Questionnaires: MADRS; CGI-S; HAMD; CGI-I; YMRS; C-SSRS; QIDS-SR; GAD-7; SDS; PRISE Neuropsychological Battery	HAMA
Sponsor/Collaborators	Ruijin Hospital	Ruijin Hospital	Ruijin Hospital	Wayne Goodman MD Baylor College of Medicine	Beijing Pins Medical Co., Ltd
Gender	All	All	All	All	All
Age	18 to 65 Years (Adult, Older Adult)	18 to 65 Years (Adult, Older Adult)	18 to 65 Years (Adult, Older Adult)	21 to 70 Years (Adult, Older Adult)	18 to 70 Years (Adult, Older Adult)
Phases	Not Applicable	Not Applicable	Not Applicable	Not Applicable	Not Applicable
Enrollment	6	13	2	6	6
Funded By	Other	Other	Other	Other	Industry
Study type	Interventional	Interventional	Interventional	Interventional	Interventional
Study designs	Allocation: N/D Intervention Model: Single Group Assignment Masking: None (Open Label) Primary Purpose: Treatment	Allocation: N/D Intervention Model: Single Group Assignment Masking: None (Open Label) Primary Purpose: Treatment	Allocation: N/D Intervention Model: Single Group Assignment Masking: None (Open Label) Primary Purpose: Treatment	Allocation: Non-Randomized Intervention Model: Sequential Assignment Masking: Quadruple (Participant, Care Provider, Investigator, Outcomes Assessor) Primary Purpose: Treatment	Allocation: N/D Intervention Model: Single Group Assignment Masking: None (Open Label) Primary Purpose: Treatment
Other study ID numbers	Habenula DBS for OCD	2018 DBS-Hb MDD	Habenula DBS	H40307 49593 277909 HSM#12-00467 GCO 12-1815	PINS-029
Start Date	March 1, 2018	November 13, 2017	July 24, 2017	February 2013	January 15, 2019
Primary completion date	February 28, 2020	July 30, 2020	November 30, 2018	August 2021	June 18, 2020
Completion date	February 28, 2020	September 30, 2020	August 30, 2019	August 2021	June 18, 2022
First posted	March 13, 2018	November 20, 2017	August 18, 2017	February 25, 2013	September 12, 2018
Results first posted	March 29, 2018	January 10, 2020	November 29, 2019	November 12, 2020	December 27, 2018
Location	Shanghai Ruijin Hospital Functional Neurosurgery, Shanghai, Shanghai, China	Shanghai Ruijin Hospital Functional Neurosurgery, Shanghai, Shanghai, China	Shanghai Ruijin Hospital Functional Neurosurgery, Shanghai, Shanghai, China	Baylor College of Medicine, Houston, Texas, United States	Shenzhen, Shenzhen, China
Investigator	N/D	N/D	N/D	Wayne Goodman MD	N/D
Responsible party	Bomin Sun, MD, PhD	Bomin Sun, MD, PhD	Bomin Sun, MD, PhD	Wayne Goodman MD	Beijing Pins Medical Co., Ltd

*BDI, Beck Depression Inventory; CGI-I, Clinical Global Impression of Improvement; CGI-S, Clinical Global Impression of Severity; C-SSRS, Columbia Suicide Severity Rating Scale; DBS, Deep Brain Stimulation; GAD-7, Generalized Anxiety Disorder 7-item Scale; GAF, Global Assessment of Functioning Scale; Hb, Habenula; HAMA, Hamilton Anxiety Scale; HAMD, Hamilton Depression Scale; MADRS, Montgomery-Asberg Depression Rating Scale; N/D, not described; SF-36, MDD, Major Depressive Disorder; MOS item short from health survey; OCD, Obsessive Compulsive Disorder; PRISE, Patient Rated Inventory of Side Effects; PSQI, Pittsburgh Sleep Quality Index; QIDS-SR, Quick Inventory of Depressive Symptomatology; Q-LES-Q-SF, Quality of Life Enjoyment and Satisfaction Questionnaire-Short Form; SDS, Sheehan Disability Scale; TRD, treatment resistant major depressive disorder; WHO-BREF, World Health Organization Quality of Life-BREF; YMRS, Young Mania Rating Scale*.

## Discussion

This literature review highlights the encouraging preliminary outcomes of Hb DBS for a variety of treatment-resistant psychiatric conditions (schizophrenia, BD, OCD, and TRD) and tentatively suggests it could be a viable therapeutic option for these conditions in the future. However, more comprehensive cases series and sham-controlled clinical trials with larger enrollment are necessary to confirm the effectiveness of Hb DBS. The review of the clinical trial databases revealed that there are a number of such trials currently underway. These larger trials will allow for a better understanding of the patient characteristics associated with greater Hb DBS benefits. For example, the authors of the Hb DBS trial for schizophrenia speculate that decreased symptom severity and shorter disease duration might play a role in determining treatment response (63). As the Hb is involved in the major neurotransmitter systems, which are the target of the most common pharmacotherapies used in these patient populations (i.e., antidepressants and antipsychotics), detailed reporting of the medication regime of patients (both before and repeatedly during Hb DBS treatment) would provide valuable information to elucidate the treatment mechanism of action. Different medication regimes as well as differences in lead localization might play a role in the great variance of DBS stimulation parameters observed in the reported studies: stimulation voltage between 1.35 and 10.5 V and frequency between 60 and 160 Hz were reported. It remains unclear if high- or low-frequency stimulation is most beneficial. For example, while Zhang and colleagues (65) report a marked improvement when switching from high- to low- frequency stimulation, Wang and colleagues (66) report good clinical outcome over 3 months with high-frequency stimulation.

Beyond the need for more additional clinical evidence, there are several challenges that should be overcome if Hb DBS is to become a useful therapeutic tool. First, greater insight into the mechanism of action of this therapy is required. This is especially important given the variety of different rationales and potential mechanisms that have been proposed for Hb DBS (Table 3). Further work with preclinical models will be needed to robustly test these proposals in the context of the various neuropsychiatric diseases. Second, given the critical relationship between the precise location and nature of the electric field and clinical response to DBS (78), knowledge of how best to target this relatively small structure and subsequently select the optimal stimulation parameters is needed. This is particularly relevant for psychiatric DBS indications given that established clinical programming algorithms of the sort employed for movement disorder patients rely largely on immediate and objective clinical feedback following parameter adjustment (e.g., improved tremor or rigidity in PD patients) (79, 80). These strategies may not be suitable for Hb DBS, where authors such as Sartorius and colleagues have reported a delay of 4 months between initiating DBS treatment and observing antidepressant effect (47). Indeed, two of the five studies report that determining the optimal stimulation parameters was time-consuming [taking 9 months for Zhang et al. (65); 8 months for Wang et al. (63)]. As such, Hb DBS–like other types of psychiatric DBS (81)–will likely benefit greatly from the identification of robust electrophysiological or neuroimaging biomarkers of efficacious stimulation. Furthermore, while most preclinical work supporting the potential use of Hb DBS in neuropsychiatric disorders has specifically focused on the function and connections of the LHb, the small size and close proximity of LHb and medial Hb make it difficult to precisely target (or visualize) only LHb in humans. Future clinical studies should therefore report DBS targeting and electrical field modeling with the utmost precision in order to evaluate the relationship between efficacy and the precise locus of stimulation within the Hb.

An array of experimental techniques may be useful for elucidating the mechanism of action and optimal treatment parameters for Hb DBS. Animal model and *in vitro* research using microelectrode recording, microdialysis, and optogenetic approaches have previously uncovered important insights into the neuronal and synaptic mechanisms of DBS for movement disorders and could be similarly applied here. Prior preclinical microelectrode recording work, for example, has shown that high frequency subthalamic stimulation leads to decreased neuronal activity in interconnected deep motor nuclei (82, 83) and suppressed local activity within the target structure (84). Microdialysis studies, which permit measurement of local neurotransmitter levels, indicate that high frequency stimulation is also accompanied by increased extracellular levels of GABA (85, 86). Optogenetic work, meanwhile, has provided further insight into the action of DBS at the ion channel level (87–89) and allowed investigations of the precise neuronal circuits underlying DBS responses (90, 91). These research modalities can be complemented by functional and molecular neuroimaging techniques such as positron emission tomography (PET), single photon emission computed tomography (SPECT), and functional magnetic resonance imaging (fMRI), which can be readily performed in patients and which capture information simultaneously from across the brain, thereby explicating the network-level effects of DBS (92–95). Indeed, recent fMRI studies have characterized brain-wide fingerprints of optimal subthalamic DBS for PD with respect to stimulation parameters such as contact, voltage, and frequency (96, 97). To this end, Zhang and colleagues used fMRI at various settings compared to a healthy control group (65) while Wang and colleagues conducted LFP recordings paired with a systematic assessment of the effects of stimulation (66).

Going forward, studies using these varying techniques will hopefully further elucidate the role of the Hb in psychiatric disease, advancing understanding of how best to modulate this structure and guiding optimization of DBS targeting and stimulation parameters. Ultimately, successful randomized, clinical trials with appropriate enrollment levels will be necessary to firmly establish the clinical benefit and optimal application of Hb DBS. Nevertheless, the reported studies (47, 63–66), albeit only reporting on a small number of patients, demonstrate that Hb DBS has potential for a multitude of clinical indications.

## Data Availability Statement

The original contributions presented in the study are included in the article/supplementary material, further inquiries can be directed to the corresponding author.

## Author Contributions

JG, MM, and AML: design of the work. JG, MM, and AT: acquisition of data. JG and MM: analysis of data and drafting the manuscript. JG, MM, GE, AL, FG, AB, and AML: interpretation of data. JG, MM, GE, AL, AT, FG, AB, and AML: critical revision of the manuscript. All authors approved the final version of the manuscript.

## Conflict of Interest

The authors declare that the research was conducted in the absence of any commercial or financial relationships that could be construed as a potential conflict of interest.

## Publisher's Note

All claims expressed in this article are solely those of the authors and do not necessarily represent those of their affiliated organizations, or those of the publisher, the editors and the reviewers. Any product that may be evaluated in this article, or claim that may be made by its manufacturer, is not guaranteed or endorsed by the publisher.
